# Choice of DNA extraction method affects detection of bacterial taxa from retail chicken breast

**DOI:** 10.1186/s12866-022-02650-7

**Published:** 2022-09-30

**Authors:** Annika Flint, Anna Laidlaw, Leo Li, Courtney Raitt, Mary Rao, Ashley Cooper, Kelly Weedmark, Catherine Carrillo, Sandeep Tamber

**Affiliations:** 1 Bureau of Microbial Hazards Health Canada, 251 Sir Frederick Banting Driveway, A.L. 2204E, Ottawa, ON K1A 0K9 Canada; 2 Canadian Food Inspection Agency, 960 Carling Road, Ottawa, ON K1A 0Z2 Canada

**Keywords:** 16S rRNA amplicon sequencing, Bacterial culture, Culture independent detection, Biotyper, DNA extraction, Metagenomics, Poultry meat

## Abstract

**Background:**

Sequence-based methods for the detection of bacteria such as 16S rRNA amplicon sequencing and metagenomics can provide a comprehensive view of the bacterial microbiome of food. These methods rely on the detection of gene sequences to indicate the presence of viable bacteria. This indirect form of detection can be prone to experimental artefacts. Sample handling and processing are key sources of variation that require standard approaches. Extracting sufficient quantities of high quality DNA from food matrices is challenging because target bacterial species are usually minor components of the microbiota and foods contain an array of compounds that are inhibitory to downstream DNA applications. Here, three DNA extraction methods are compared for their ability to extract high quality bacterial DNA from retail chicken breast rinses, with or without enrichment. Method performance was assessed by comparing ease of use, DNA yield, DNA quality, PCR amplicon yield, and the detection of bacterial taxa by 16S rRNA amplicon sequencing.

**Results:**

All three DNA extraction methods yielded DNA of sufficient quantity and quality to perform quantitative PCR and 16S rRNA amplicon sequencing. The extraction methods differed in ease of use, with the two commercial kits (PowerFood, PowerSoil) offering considerable time and cost savings over a hybrid method that used laboratory reagents for lysis and commercial column based kits for further purification. Bacterial richness as determined by 16S rRNA amplicon sequencing was similar across the three DNA extraction methods. However, differences were noted in the relative abundance of bacterial taxa, with significantly higher abundance of Gram-positive genera detected in the DNA samples prepared using the PowerFood DNA extraction kit.

**Conclusion:**

The choice of DNA extraction method can affect the detection of bacterial taxa by 16S rRNA amplicon sequencing in chicken meat rinses. Investigators should be aware of this procedural bias and select methods that are fit for the purposes of their investigation.

**Supplementary Information:**

The online version contains supplementary material available at 10.1186/s12866-022-02650-7.

The bacterial microbiome of meat is a dynamic collection of organisms that changes in response to multiple factors encountered during the rearing and processing of food animals. These bacterial populations are diverse and interact with each other and their environment. In addition to environmental species and animal commensals, the meat microbiome may also include priority species such as spoilage organisms, lactic acid producing bacteria, hygiene indicators, and pathogens [1]. The impact these bacteria have on human health, food quality, and food safety underscores the importance of reliable detection, identification, and quantification of bacteria in foods.

Bacterial species of economic or safety concern may not be the largest proportion of the microbiota. Pathogens, particularly may be present in foods at low numbers [2]. Therefore, procedures for their detection often involve a non-selective enrichment period wherein the analytical unit is incubated in microbiological broth media. This enrichment period allows bacteria to recover from stress and injury and grow to detectable levels. Traditional detection methods employ 18 – 24 h enrichment periods followed by plating onto solid agar media for colony isolation and identification. These methods are tailored to specific taxa of interest and thus offer a narrow view of the types of bacteria present in meat. Additionally, the methods may select for the most robust, typical strains of a genus. Injured or stressed cells and unusual variants can be missed. Rapid methods forgo plating onto agar for the molecular detection of specific pathogen moieties (e.g., DNA sequence for PCR, surface antigens for ELISA). However, these methods also require an enrichment period in broth to reach the sensitivity (e.g., one cell in a 25 g analytical unit) required to make regulatory decisions [3].

Sequence based detection methods such as metagenomics and 16S rRNA amplicon sequencing use nucleic acids extracted directly from samples of interest. The DNA is subject to sequencing and bacteria are identified based on the detection of specific sequences in the pool of total DNA. This approach offers comprehensive but indirect presence/absence testing at high levels of resolution. In the field of food microbiology, sequence-based detection tools hold the promise of analyzing the entire bacterial community structure of foods without the need for extensive culturing. Forgoing this requirement would enable the detection of multiple species per sample, including difficult to detect bacteria, injured cells, VBNC cells, unusual variants, and multiple strains of the same species [4].

With routine sequencing runs approaching 50 million reads, detection of bacterial sequences enables in-depth analysis of a microbial community, but the method is prone to bias and experimental artifacts. Study design parameters such as DNA extraction methods, sample handling, and sequencing methodology can affect the levels and types of sequence reads detected [5, 6]. To date, DNA extraction protocols for microbiome analyses have largely focused on environmental and clinical samples [7, 8] and data on food matrices, particularly meat, is lacking. Food matrices present unique challenges to the extraction of high-quality DNA for downstream analysis. Gene amplification reactions by PCR can be inhibited by high concentrations of protein and fat [9]. Additionally, the presence of low numbers of target species such as pathogens in meat often requires enrichment to reach detectable levels [2, 10]. Typically, cultures are enriched overnight (18 – 24 h) to enable growth and recovery of target organisms. However, with some rapid detection methods, a shorter 6 h enrichment is possible [10]. Even with enrichment, certain media components may interfere with the activity of enzymes needed for molecular analyses [9]. Additionally, the presence of high abundance taxa can mask the presence of sparse communities due to inefficient growth, or poor lysis of cells. Therefore, the objective of this study was to compare the performance of three DNA extraction methods on the detection of bacteria taxa in retail chicken breast surface rinses without enrichment or enriched for 6 or 18 h. Chicken meat is an important source of bacterial pathogens such as *Campylobacter* and *Salmonella*, and poultry rinses are often targeted for food safety monitoring programs [1]. The DNA extraction methods were chosen based on their ability to extract DNA from difficult sample matrices. Performance was assessed based on ease of use, DNA yield, DNA quality, PCR amplicon yield, and the composition of the chicken breast microbiome as determined by 16S rRNA amplicon sequencing.

## Materials and methods

### Chicken breast samples and sampling procedure

**Fig. 1:**
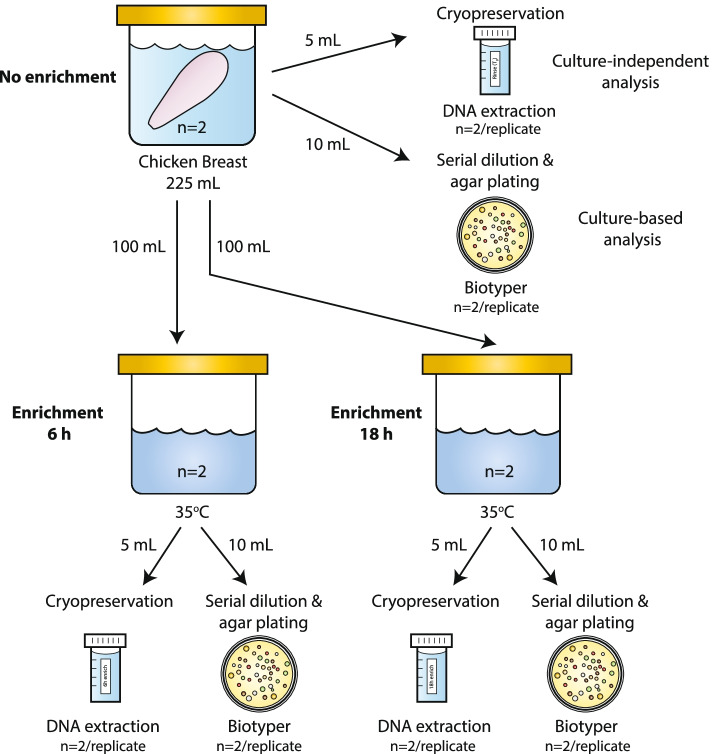
Flow diagram illustrating the sample handling and analysis of chicken breast samples. Two fresh boneless, skinless chicken breasts taken from the same retail package were used in this study (*n* = 2). Each chicken breast was rinsed in buffered peptone water (BPW). The rinsate was divided into three portions and each was incubated as indicated (no enrichment, 6 h enrichment, and 18 h enrichment). Samples from each portion and enrichment time were removed for cryopreservation and agar plating. Culture independent analysis was performed with the no enrichment cryopreserved sample. Culture-based analysis involved culturing the rinsate in broth followed by plating on agar and colony identification using a Biotyper. Broth analysis involved collecting the cells following enrichment for DNA extraction and 16S rRNA analysis

Two boneless, skinless chicken breasts packaged in the same retail pack were used for this study. To mimic conditions in a routine testing lab and to prevent the growth of spoilage organisms, chicken breasts were stored at -20 °C after purchase and thawed at 4 °C the night preceding the experiment. A rinsate of each chicken breast was prepared by placing each breast in a sterile sampling bag and submerging in 225 mL buffered peptone water (BPW; Oxoid, Nepean, ON, Canada). Samples were thoroughly mixed by massaging and shaking intermittently for 1 min. Each sample was portioned as follows: 100 mL of rinsate was incubated at 35 °C for 6 h, 100 ml of rinsate was incubated 35 °C for 18 h, the remaining rinsate was used as the no enrichment sample (0 h) (Fig. 1). At each time point (0 h, 6 h, and 18 h), aliquots of each rinse/broth culture were serially diluted and plated in duplicate on plate count agar (PCA, Oxoid) for total aerobic mesophilic bacteria enumeration results and bacterial colony identification. Equal portions (5 mL) of each rinse/broth culture were removed and snap-frozen in liquid N2 and stored short-term at -80 °C prior to DNA extraction.

### Bacterial colony identification

For the culture-based detection, all of the colonies from the PCA plates from each time point (no enrichment, 6 h enrichment, and 18 h enrichment) that had between 30 – 300 CFU/plate were identified. A benchtop matrix-assisted time-of flight mass (MALDI-TOF) spectrometer (Bruker Daltonics GmbH & Co. KG, Milton, ON, Canada) was used as described previously using MALDI Biotyper® 3.1 and BDAL v. 9.0.0 (8468) [11]. Reported results are of colonies with high-confidence identification scores (≥ 1.70). Colonies with lower confidence identification scores (< 1.70) are reported as unidentified [12]. Relative abundances were calculated as the percent of colonies of each genus out of the total number of colonies analyzed. Results from both chicken breasts were combined and reported as one.

### DNA extraction

Total DNA was extracted from each chicken breast sample after three enrichment time points (0 h, 6 h, and 18 h) in duplicate. The performance of two commercially available DNA extraction kits (DNeasy PowerFood and DNeasy PowerSoil, both from Qiagen, Germantown, MD) was compared to a hybrid method [13]. Briefly, the hybrid method used laboratory reagents and zirconia beads to achieve cell lysis through chemical and mechanical means. The released DNA was precipitated using ethanol and purified further using the QIAamp DNA Stool Mini Kit (Qiagen) and the OneStep PCR Inhibitor Removal Kit (Zymo-Research Corp., Irvine, CA). Purified DNA samples were quantitated using a Qubit 4 fluorometer and a broad range nucleic acid kit (Thermo Fisher Scientific, Ottawa, ON, CA). Quality assessment was based on A260/A280 using a Nanodrop UV spectrophotometer for DNA purity (Thermo Fisher Scientific, Ottawa, ON, CA) and appearance on a 0.8% agarose-TBE gel for DNA integrity.

### Quantitative PCR

The ratio of bacterial (16S rRNA rRNA) and host (18S rRNA) DNA was determined by quantitative PCR (qPCR) using the primers listed in Table 1 [14, 15]. qPCR master mixes consisted of 1X SsoAdvanced Universal SYBR Green Supermix (Bio-Rad Laboratories, Saint-Laurent, QC, CA), 1.0 µM primers, 0.2 µM probe, and 1 ng template per qPCR reaction. Cycling was done in a CFX96 Real time PCR Detection system (Bio-Rad) at 95 °C for 10 min, followed by a 2 step setting of 95 °C for 15 s and 60 °C for 60 s for 45 cycles. Standard curves were constructed using tenfold serial dilutions of *Cryptosporidium parvum* oocyst DNA (18S rRNA, Waterborne, Inc.) and a pooled aliquot of all of the DNA samples extracted for this study (16S rRNA). Standard curve analysis and amplicon quantitation were carried out using CFX Manager v. 3.1 (Bio-Rad).

**Table 1:** 16S rRNA rRNA and 18S rRNA qPCR primers and probes used in this study

Primer/Probe	Target	Sequence /reporter dye/	Reference
Euk1141F	18S rRNA	GAATTGACGGAAGGGCACCAC	15
Euk1440R	18S rRNA	GGCATCACAGACCTGTTAT	15
Euk1266-Probe	18S rRNA	/56-FAM/TG GTG GTG C/ZEN/A TGG CCG TTC TT/3IABkFQ/	15
BactQF	16S rRNA rRNA	CCTACGGGDGGCWGCA	14
BactQR	16S rRNA rRNA	GGACTACHVGGGTMTCTAATC	14
BactQ-Probe	16S rRNA rRNA	/5HEX/CA GCA GCC G/ZEN/C GGT A/3IABkFQ/	14

### 16S rRNA amplicon sequencing

16S rRNA amplicon sequencing libraries were constructed using the Quick-16S rRNA NGS Library Prep Kit and Quick-16S rRNA Primer Set V3-V4 following the High Microbial DNA protocol (Zymo Research Corp, Irvine, CA, USA) using 20 ng input DNA. Paired-end Illumina sequencing was performed on a MiSeq instrument with kit v3 chemistry, 2 × 300 bp as specified by the manufacturer (Illumina Inc., San Diego, CA, USA).

### 16S rRNA amplicon read processing and taxonomic classification

Reads were processed using Cutadapt v2.4 [16] to remove adapter and barcode sequences by length (-u 16 or 24 flag for forward and reverse reads respectively), and trim 3’ end low quality nucleotides (using -q 20 flag). Paired-end reads were merged using Pear v0.9.6 with minimum length (-n 100) and quality threshold (-q 20) commands, [17] and fed into QIIME 2 v2019.7.0 [18] for further processing and OTU picking. Denoising, chimera removal and dereplication of reads were performed with Deblur [19] and the qiime deblur denoise-other function within QIIME 2 using the 90% similarity SILVA database (release 132: 10/04/2018, Quast et al., 2013) and –p-trim-length 350. Closed reference OTU similarity clustering and taxonomic assignment were performed using the QIIME 2 vsearch cluster-features-closed-reference and feature-classifier classify-consensus-vsearch commands and the SILVA database (release 132: 10/04/2018) at 97% similarity.

### 16S rRNA microbial community analysis

Phyloseq v1.34 [20] was used to rarefy OTUs to 5000 reads/sample, calculate alpha and beta diversities, and determine relative abundances of taxa within samples. Samples were rarefied using the rarefy_even_depth command with a sample size of 5000, random seed of 31 and replace = false). Alpha diversities were measured using Chao1 (genus richness) and Shannon (genus diversity) indexes (estimate_richness function using default parameters). Microbial community composition was determined using principal coordinate analysis (PCoA) on the weighted UniFrac distances (ordinate function using PCoA, unifrac and weighted = true options). Taxonomic changes over time for each kit were analyzed using DESeq2 v1.12.3 [21] (using test = wald and fittype = parametric) with fold changes > 1.5 and false discovery rate adjusted *P* values < 0.05 considered significant [22]. Data was graphed using Graph Pad Prism except for PCoA plots, which were graphed using R package ggplot2 (v3.3.3, https://CRAN.R-project.org/package=ggplot2).

### Statistical analysis

Unless stated otherwise, reported values are the mean ± standard deviation result of two technical repeats using two unique chicken breast samples (*n* = 4). Significant differences between methods were determined by one-way analysis of variance (ANOVA) with the Tukey post-hoc test using Graph Pad Prism v 9.

## Results

### Culture-based characterization of the retail chicken breast microbiome

**Fig. 2:**
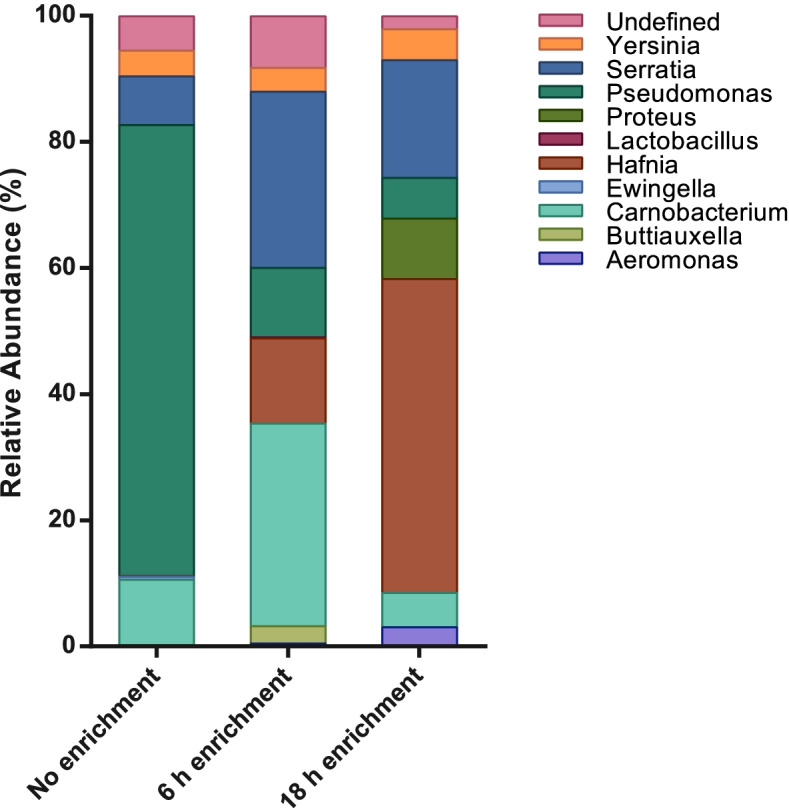
Average relative abundances of genera identified on retail chicken breast as determined by the Bruker Biotyper. Taxa abundances of identified colonies at the genus level are shown for each enrichment condition. Undefined: colonies with identification scores < 1.70 using the BDAL Bruker database (Version V9.0.0 8468)

The total aerobic mesophilic bacteria enumeration result indicated that the level of bacteria in the chicken breast rinsates increased from 5.5 ± 0.1 log CFU/mL with no enrichment (0 h) to 6.8 ± 0.1 log CFU/mL and 8.7 ± 0.2 log CFU/mL after 6 h and 18 h of incubation in BPW at 35 °C, respectively. The identities of the recovered colonies are shown in Fig. 2 (genus-level) and Supplementary Fig. 1 (species-level). Overall, the composition of the bacterial microbiota was similar between the two chicken breasts and data are presented as a composite of the two samples. In total, 1417 colonies were selected for identification and 93% yielded an unambiguous identification using the Bruker Biotyper. Thirty-seven bacterial species belonging to eleven genera, nine families, and five orders were identified (Fig. 2 and Supplementary Fig. 1). In the absence of enrichment, the culturable microbiota was dominated by *Pseudomonas* with an average relative abundance of 71%. After 6 h of enrichment *Carnobacterium* and *Serratia* predominated with respective relative abundances of 32% and 28%. *Hafnia* was the predominant genus after the 18 h enrichment with a relative abundance of 50%. *Serratia* and *Proteus* were also predominant at 18 h with relative abundances of 19% and 10% respectively. More sparse but notable genera identified included *Aeromonas* (0.4% at 6 h and 3% at 18 h), *Lactobacillus* (0.2% at 6 h) and *Yersinia* (~ 4% across all times points).

The composition of the total aerobic microbiota shifted upon prolonged enrichment. The relative abundance of *Pseudomonas* decreased with incubation time (from > 70% at 0 h to 11% and 6% relative abundance at 6 h and 18 h, respectively). The relative abundance of *Carnobacterium* peaked at 6 h to 32% and then decreased to 5% after the overnight incubation. Members of the order *Enterobacterales* increased in relative abundance and richness as the enrichment progressed: three genera (*Hafnia*, *Serratia*, and *Yersinia*) were identified at 0 h with a combined relative abundance of 12% and by 18 h a combined relative abundance of 83% was observed, and the number of *Enterobacterales* genera identified expanded to include *Proteus*.

### Comparison of DNA extraction kits: Performance metrics

The three DNA isolation methods used in this study relied on a combination of chemical and mechanical lysis to disrupt cells. The primary difference among the three was the method of DNA collection and inhibitor removal. Both the PowerFood and PowerSoil kits collected the DNA through adsorption to a silica column, with multiple washes to remove inhibitory compounds prior to DNA elution. The hybrid method relied on DNA precipitation and RNA/protein removal prior to column adsorption and DNA washing. The eluted DNA was subjected to another column adsorption/washing procedure to remove PCR inhibitory reagents. In terms of A260/A280 readings, the DNA obtained using all three methods exceeded 1.8 and minimal shearing was observed by gel electrophoresis (data not shown).

All of the extraction methods yielded sufficient DNA to perform downstream analysis with amounts ranging from 1365 to 8620 ng (Table 2). No significant differences in DNA quantity were observed among the kits at the earlier time points, however, higher levels of DNA were recovered at 18 h with the PowerSoil kit compared to 0 h and 6 h (*P* < 0.05). Also at 18 h, both the PowerSoil and hybrid method yielded approximately three-fold higher concentrations of DNA compared to PowerFood (*P* < 0.05). There was a considerable amount of variation in the concentration of DNA recovered between the replicates, particularly with the hybrid method. The coefficients of variation ranged from 0.8% (PowerFood, 0 h) to 73% (hybrid method, 6 h).

**Table 2:** DNA yield (mean ng ± SD) obtained using three DNA extraction methods

Time	PowerFood	PowerSoil	Hybrid
0 h	1680 ± 14	1785 ± 587	3840 ± 1556
6 h	1655 ± 7718	1365 ± 473	1791 ± 1299
18 h	2135 ± 318^a^	8620 ± 990^b^	6540 ± 127

^a^significantly lower (*P* < 0.05) than PowerSoil and Hybrid as determined by one-way analysis of variance (ANOVA) with the Tukey post-hoc test, *n* = 4

^b^significantly higher (*P* < 0.05) than 0 h and 6 h, as determined by one-way analysis of variance (ANOVA) with the Tukey post-hoc test, *n* = 4

The PowerFood kit was the most economical DNA extraction method, costing a little over $5 an extraction and taking 34 min from cell pellet to purified DNA (Table 3). The hybrid method, which required two DNA columns, was the most expensive and time-consuming at $10.28 per extraction and a 2 h 10 min extraction time. The extraction method did not significantly affect the number of OTUs detected by 16S rRNA amplicon sequencing but more OTUs were detected at 18 h compared to the earlier time points (Table 3).

**Table 3:** Resource requirements and outputs of three DNA extraction methods

Method	Cost per extraction^a^	Extraction time	Number of OTUs detected		
			0 h	6 h	18 h
PowerFood	$ 5.02	34 min	14 ± 1	13 ± 1	18 ± 2^b^
PowerSoil	$ 8.27	36.5 min	14 ± 1	14 ± 1	17 ± 2
Hybrid	$ 10.28	2 h 10 min	13 ± 1	13 ± 1	19 ± 3^c^

^a^cost reflects pricing available to Canadian government laboratories at the time the study was conducted

^b^significantly higher (*P* < 0.05) than at 0 h and 6 h, as determined by one-way analysis of variance (ANOVA) with the Tukey post-hoc test, *n* = 4

^c^significantly higher (*P* < 0.05) than at 0 h and 6 h, as determined by one-way analysis of variance (ANOVA) with the Tukey post-hoc test., *n* = 4

**Fig. 3:**
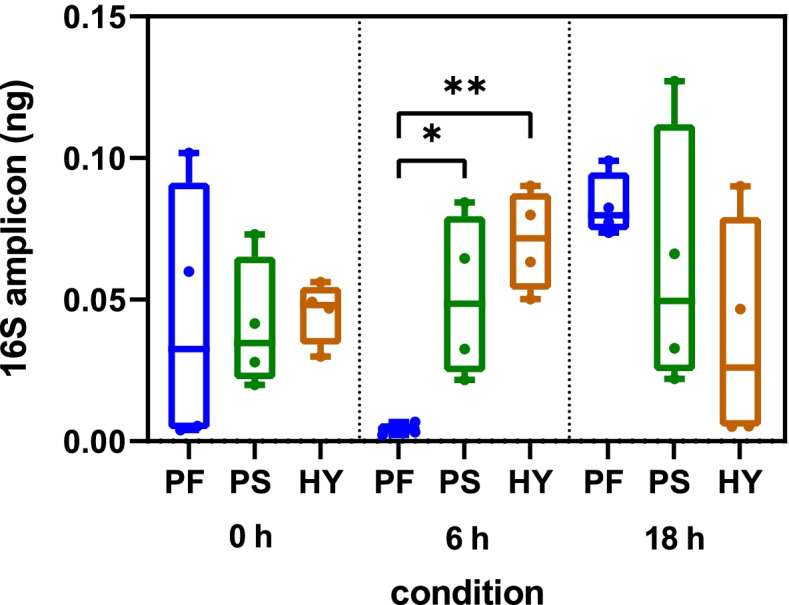
Quantification of 16S rRNA using three different DNA extraction kits. DNA was extracted from retail chicken breast at three enrichment timepoints (0 h, 6 h, 18 h) using the PowerFood, PowerSoil and Hybrid DNA extraction kits followed by qPCR. Horizontal lines represent the median; boxes indicate the inter-quantile range and whiskers represent the maximum and minimum values of the dataset. Asterisks denote statistically significant differences (*P ≤ 0.05, **P ≤ 0.01) using one-way analysis of variance (ANOVA) with the Tukey post-hoc test. PF: PowerFood; PS: PowerSoil and HY: Hybrid

All of the DNA samples were able to support the amplification of a 16S rRNA rRNA gene fragment with qPCR efficiencies of 90%—98% and threshold cycles ranging from 16 to 22 (data not shown). No significant differences were noted in the amount of 16S rRNA amplicon obtained with each DNA sample at 0 h and 18 h. However, the DNA extracted using the PowerFood kit yielded significantly lower levels of 16S rRNA rRNA amplicon at 6 h (Fig. 3, *P* < 0.05). The DNA samples contained a very low level of 18S rRNA with threshold cycles ranging from 25 to 36 corresponding to 0.08 – 1.3 fg 18S amplicon (data not shown).

### Comparison of DNA kits: Microbiome compositional analysis

To investigate the impact of DNA extraction kit on microbial composition, richness and diversity, samples were analyzed by 16S rRNA rRNA next generation sequencing targeting the V3-V4 hypervariable region. Sequencing yielded ~ 7 million raw reads with a median of 146,664 reads per sample (*n* = 36). Following denoising, dereplication and chimera removal, samples had a median of 11,141 reads. One PowerFood sample (no enrichment) was excluded from analysis due to low read counts.

**Fig. 4:**
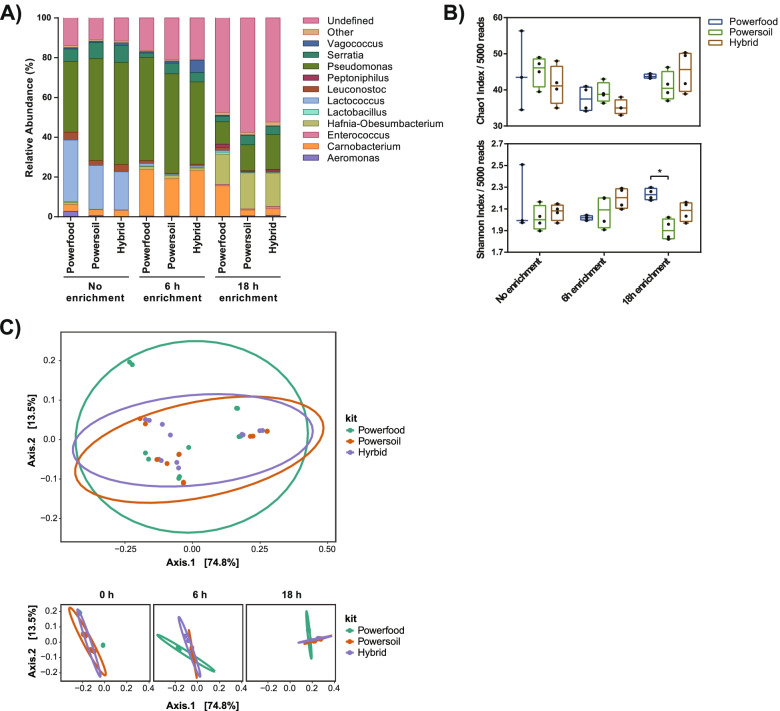
Relative abundances, alpha and beta diversities of DNA extraction kits at each enrichment time point. **A** Average relative abundances of genera are similar between DNA extraction kits at each time point. Taxa abundances of 16S rRNA sequences at the genus level are shown for each kit by sampling time point. Relative abundances are shown for genera > 1% in at least 1 sample. Undefined: OTUs that could not be identified down to the genus level; Other: OTUs present at < 1% were grouped and summed. **B** Genus diversity, but not richness, is altered by choice of DNA extraction kit. Chao1 index (upper panel) and Shannon index (lower panel) of chicken breast 16S rRNA microbiota by enrichment time point. Horizontal lines represent the median, boxes indicate the inter-quantile range and whiskers represent values within 1.5 IQR of the lower and upper quantiles. Alpha diversities were calculated using PhyloSeq in R. Asterisks denote statistically significant differences (*P* < 0.05) using t-tests. **C** Microbial composition is not altered by DNA extraction kit. Principal co-ordinate analysis of weighted Unifrac distances for all 16S rRNA samples (upper panel) and 16S rRNA samples by enrichment time (lower panel). Ellipses represent 95% confidence regions

Overall, 16S rRNA amplicon sequencing identified 25 genera in at least one of the kits in at least one time point (Table 4). All 10 genera identified by Bruker, except for *Ewingella* and *Buttiauxella,* were identified by 16S rRNA sequencing. With no sample enrichment, the microbial abundance profiles between DNA extraction kits were similar with *Pseudomonas* (36–51%), *Lactococcus* (19–31%), and *Serratia* (6–9%) being the most abundant taxa (Fig. 4A). The PowerFood kit was better at detecting lower abundance taxa such as *Aeromonas* and *Hafnia* at 2.7% and 1.2%, respectively, compared to the PowerSoil and hybrid methods. At 6 h of enrichment, all DNA extraction kit samples showed similar relative abundance of genera with *Pseudomonas* (41 – 51%) and *Carnobacterium* (19 – 23%) as the most abundant taxa observed. The hybrid method performed significantly better for detection of the food spoilage organism *Vagococcus* in contrast to the other kits (Table 4, 6% vs 0.6 and 1%, *P* < 0.05). At 18 h of enrichment, the most predominant group for each extraction kit belonged to genera that could not be defined down to the genus level using the 16S rRNA SILVA database (undefined 48 – 58%) but were classified as *Enterobacteriaceae* at the family taxonomic level. Between the kits at 18 h, the PowerFood samples were significantly more abundant for *Carnobacterium, Lactobacillus and Leuconostoc* (*P* < 0.05, Table 4). The PowerSoil and hybrid method samples had more similar taxonomic abundance profiles overall and performed significantly better for detection of *Serratia* (*P* < 0.05, Table 4).

**Table 4:** Mean relative abundances of 16S rRNA rRNA samples for each DNA extraction method and enrichment time

Genus	PowerFood			PowerSoil			Hybrid		
	**0 h**	**6 h**	**18 h**	**0 h**	**6 h**	**18 h**	**0 h**	**6 h**	**18 h**
**Gram-positive**									
*Brochothrix*	0.71	**0.32**^**b**^	0.46	0.65	0.11	0.14	0.72	0.06	0.38
*Carnobacterium*	3.46	23.43	**15.38**^**ab**^	2.95	18.82	2.93	3.20	22.88	3.36
*Clostridium *sensu stricto* 7*	-	-	0.02	-	-	0.06	-	-	0.01
*Clostridium *sensu stricto* 18*	-	-	-	-	-	-	-	-	0.03
*Enterococcus*	-	-	0.61	-	-	0.52	-	-	1.18
*Gallicola*	-	-	0.07	-	-	-	-	-	0.01
*Hathewaya*	-	-	0.35	-	-	0.77	-	-	0.73
*Lactobacillus*	**0.26**^**a**^	0.30	**1.10**^**ab**^	**0.08**^**b**^	0.19	0.18	0.26	0.28	0.23
*Lactococcus*	30.99	**1.25**^**ab**^	0.78	21.96	0.52	0.20	19.14	0.73	0.26
*Leuconostoc*	**4.00**^**a**^	**1.51**^**a**^	**1.60**^**a**^	2.38	0.30	0.32	3.68	0.84	0.49
*Peptoniphilus*	-	-	1.57	-	-	0.30	-	-	0.75
*Vagococcus*	0.58	**0.61**^**b**^	0.50	0.64	**1.02**^**b**^	0.07	1.20	6.13	0.13
**Gram-negative**									
*Acinetobacter*	-	0.01	0.01	0.01	0.01	-	0.01	-	0.02
*Aeromonas*	2.67	0.32	0.21	0.60	0.33	0.45	-	0.29	0.72
*Comamonas*	-	-	0.01	-	-	0.06	-	-	0.01
*Escherichia-Shigella*	-	-	0.21	-	-	0.21	-	-	0.40
*Hafnia*	1.19	1.48	**15.26**^**a**^	0.24	1.67	18.20	0.01	1.36	16.76
*Janthinobacterium*	0.02	0.02	0.01	0.01	-	-	0.01	-	0.01
*Morganella*	0.02	0.01	-	0.02	0.01	0.01	0.03	0.01	0.02
*Myroides*	-	-	-	-	-	-	0.01	-	-
*Proteus*	0.01	-	0.09	0.02	-	0.06	-	-	0.23
*Providencia*	0.01	0.01	-	0.05	0.02	-	0.02	0.01	-
*Pseudomonas*	35.59	51.84	11.35	51.39	50.06	13.16	51.40	41.48	17.61
*Serratia*	6.29	**2.30**^**ab**^	**2.78**^**ab**^	8.10	5.41	4.83	8.63	4.69	4.23
*Yersinia*	0.26	0.21	0.09	0.12	0.44	0.03	0.11	0.31	0.13

^a^significantly different to PowerSoil at this timepoint, *P* < 0.05; Significant differences were determined using DESeq2 with fold changes > 1.5 and false discovery rate adjusted P values

^b^significantly different to the hybrid method at this timepoint, *P* < 0.05;

-not detected in the rarefied count table. Significant differences were determined using DESeq2 with fold changes > 1.5 and false discovery rate adjusted *P* values < 0.05 considered significant, *n* = 4

Analysis of genus richness (Chao1 index) revealed no significant differences between DNA extraction kit samples for each enrichment time point (Fig. 4B). A higher level of genera evenness (Shannon diversity, a measure of how close in abundance different taxa are) was observed with the PowerFood kit compared to the PowerSoil kit at 18 h enrichment (*P* < 0.05, Fig. 4B).

Principal coordinate analysis (PCoA) of β-diversity using weighted Unifrac was performed to profile microbial community composition. No significant separation was observed by kit type (Fig. 4C); however, samples did cluster separately by time point (Supplementary Fig. 2). Specifically, the 18 h samples show clear separation and distinct microbial composition compared to the no enrichment and 6 h samples.

Comparison of the 16S rRNA sequencing data to that obtained by agar plate culturing and Bruker identification, showed similarities at each enrichment time but also key differences. Specifically, 16S rRNA rRNA sequencing identified 15 more genera than the culture-based method. The agar plate culturing method did not yield growth of Gram-negative taxa such as *Acinetobacter*, *Comamonas*, *Escherichia-Shigella*, *Janthinobacterium*, *Morganella*, *Myroides*, or *Providencia*, which were detectable by 16S rRNA. These organisms are well known poultry meat microbiota with many involved in spoilage [23]. Furthermore, 16S rRNA was able to identify the Gram-positive genera *Brochothrix*, *Clostridium*, *Enterococcus*, *Gallicola*, *Hathewaya*, *Lactococcus*, *Leuconostoc*, *Peptoniphilus*, and *Vagococcus.* While *Lactococcus* was detected at a high abundance (> 19%) by 16S rRNA amplicon sequencing, it was not recovered using the culturing conditions of this study.

## Discussion

Sequence-based methods for the identification of bacterial taxa have the potential to offer a more comprehensive view of the food microbiota compared to culture-based methods. However, these methods are prone to bias and require consistent approaches to achieve reproducibility between samples and studies. Here we report the impact of different DNA extraction methods on the detection of naturally occurring bacterial taxa in rinses prepared from retail chicken breast.

The three DNA extraction methods were chosen based on their ability to extract DNA from difficult sample types, such as food. All three methods used a combination of chemical and mechanical disruption for cell lysis. The composition of the lysis solutions is not known for the commercial kits, and differences here may have biased the efficiency of lysis towards certain taxa. The primary difference between methods was in how inhibitory compounds were removed. Both PowerFood and PowerSoil used proprietary solutions to remove the inhibitors, whereas the hybrid method employed an additional column spin kit, adding considerable expense to the extraction method. The DNA precipitation step of the hybrid method added considerable time to the purification procedure and did not result in substantially greater yields of DNA. Differences in DNA yield among the extraction procedures were of minor consequence to our study since in all cases sufficient quantities of DNA were extracted for qPCR, and 16S rRNA amplicon sequencing. It was noted that the DNA yield with the hybrid method was more variable than with the commercial kits, suggesting that its outcome is more sensitive to technical variation and the commercial kits offer a more standardized approach.

DNA quality as assessed by A260/280 ratio, gel electrophoresis, and amount of host (18S) DNA, was equivalent across extraction methods. A difference in the yield of 16S rRNA amplicon was noted with the 6 h PowerFood DNA compared to hybrid method and PowerSoil DNA samples. This lower yield may have been due to the presence of inhibitory compounds in this particular sample. As a result, the lower amount of 16S rRNA may have influenced the subsequent detection of bacterial genera and could explain why the evenness of the bacterial community was significantly lower with the 6 h PowerFood DNA compared to the hybrid method DNA, despite both methods yielding an equivalent number of OTUs [13].

Compared to the traditional culture-based method, a greater diversity of taxa, both Gram-negative (*Acinetobacter*, *Comamonas*, *Escherichia*-*Shigella*, *Janthinobacterium*, *Morganella*, *Myroides*, or *Providencia*) and Gram-positive (*Brochothrix*, *Clostridium*, *Enterococcus*, *Gallicola*, *Hathewaya*, *Lactococcus*, *Leuconostoc*, *Peptoniphilus*, and *Vagococcus*) was detected with 16S rRNA amplicon sequencing. Although many of these genera have been reported in chicken meat and are of interest for food production, most have little clinical relevance and may not be represented in the Bruker database [1, 23]. Additionally, given the indirect nature of 16S rRNA amplicon sequencing, it may be possible that some genera were not present in our chicken breast samples as viable bacteria, or were present at levels below the detection limit of standard plate culture (log 2 CFU/g) even after an 18 h enrichment period. Two genera, *Ewingella* and *Buttiauxella*, were identified with the culture-based method but not by 16S rRNA amplicon sequencing. This result could have been due to deficiencies in the SILVA database or the amount of sequence data analyzed; a deeper sequencing approach may have been able to detect these low abundance taxa. Alternatively, the lack of detection of certain genera could be a reflection of DNA extraction, amplification and/or sequencing bias that can lead to false negative results.

The use of an enrichment procedure had a considerable impact on the number and relative abundance of bacterial taxa detected. More taxa were recovered after an 18 h enrichment, and the population shifted from the indigenous populations expected to reside on chicken meat (*Pseudomonas*, *Lactococcus*, *Carnobacterium*) to members of the order *Enterobacterales* [1, 23]. Enrichment procedures can also lead to bias, especially towards highly abundant taxa, or those with robust growth phenotypes. Thus, the choice to enrich depends on whether the target organism is abundant in the matrix or has the capacity to out-compete other taxa in the chosen enrichment conditions.

Comparison of the bacterial community structure by 16S rRNA amplicon sequencing revealed that the relative abundance of many Gram positive genera were higher using the PowerFood DNA compared to the other two DNA preparations, resulting in a more even bacterial community. The communities detected using either PowerSoil or hybrid method DNA trended towards higher relative abundances of Gram-negative genera, which was more in line with the results of the culture-based method. Given the small sample size used in this study, no firm conclusions can be drawn on the composition of the chicken breast microbiome. Further work is required to define the balance of Gram-negative and Gram-positive genera in this matrix. Differences in the recovery of Gram positive organisms based on extraction method have also been reported for other matrices [24].

The results of our study demonstrate that there are small but important differences among DNA extraction methods used to isolate DNA from retail chicken meat. These results, along with others, emphasize the importance of sample preparation when investigating the microbiome of food products [25, 26]. Inaccurate detection of the food microbiome, or specific taxa within it, has the potential to impact food quality and food safety assessments. In terms of ease of use, expense, and evenness of the bacterial community structure, the performance of the PowerFood kit was superior in our study. However, given the small number of chicken breast samples analyzed, and differences among food matrices, we cannot make a blanket recommendation for all food types and suggest smaller fit-for-purpose studies such as this before proceeding with a large-scale analysis.

## Conclusions

The DNA extraction method is a key variable to consider when using sequence-based tools to detect bacteria in foods. A study aiming to characterize the entire microbiome of a food sample may require a different approach that prioritizes DNA extraction from all taxa (e.g., PowerFood) than one focussed on the detection of a smaller number of key taxa (e.g., PowerSoil, or a more tailored hybrid approach). The results of this study do not necessarily allow, but encourage researchers to evaluate their DNA extraction and enriching protocols for each type of matrix before attempting to use them for detection purposes. Many inhibitors exist depending on the matrix, in this case, chicken breast rinses appeared not to have many PCR inhibitors.

## Supplementary Information

**Additional file 1:** **Supplementary Figure 1. **Relative abundances of species identified on retail chicken breast as determined by the Bruker Biotyper.

**Additional file 2:** **Supplementary Figure 2. **Microbial composition is altered by enrichment time.

## Data Availability

Reads and metadata from 16S rRNA metagenomics and WGS metagenomics sequencing have been deposited to the National Center for Biotechnology Information Sequence Read Archive under BioProject accession PRJNA721708 (https://www.ncbi.nlm.nih.gov/bioproject/?term=721708).
