# Supplementary material for: Choice of DNA extraction method affects detection of bacterial taxa from retail chicken breast
Source: BMC Microbiol. 2022 Sep 30;22:230. doi: 10.1186/s12866-022-02650-7 (PMC9524001; doi:10.1186/s12866-022-02650-7)
Supplement: Supplementary file 1 — Additional file 1: Supplementary Figure 1. Relative abundances of species identified on retail chicken breast as determined by the Bruker Biotyper. [file 12866_2022_2650_MOESM1_ESM.pdf]

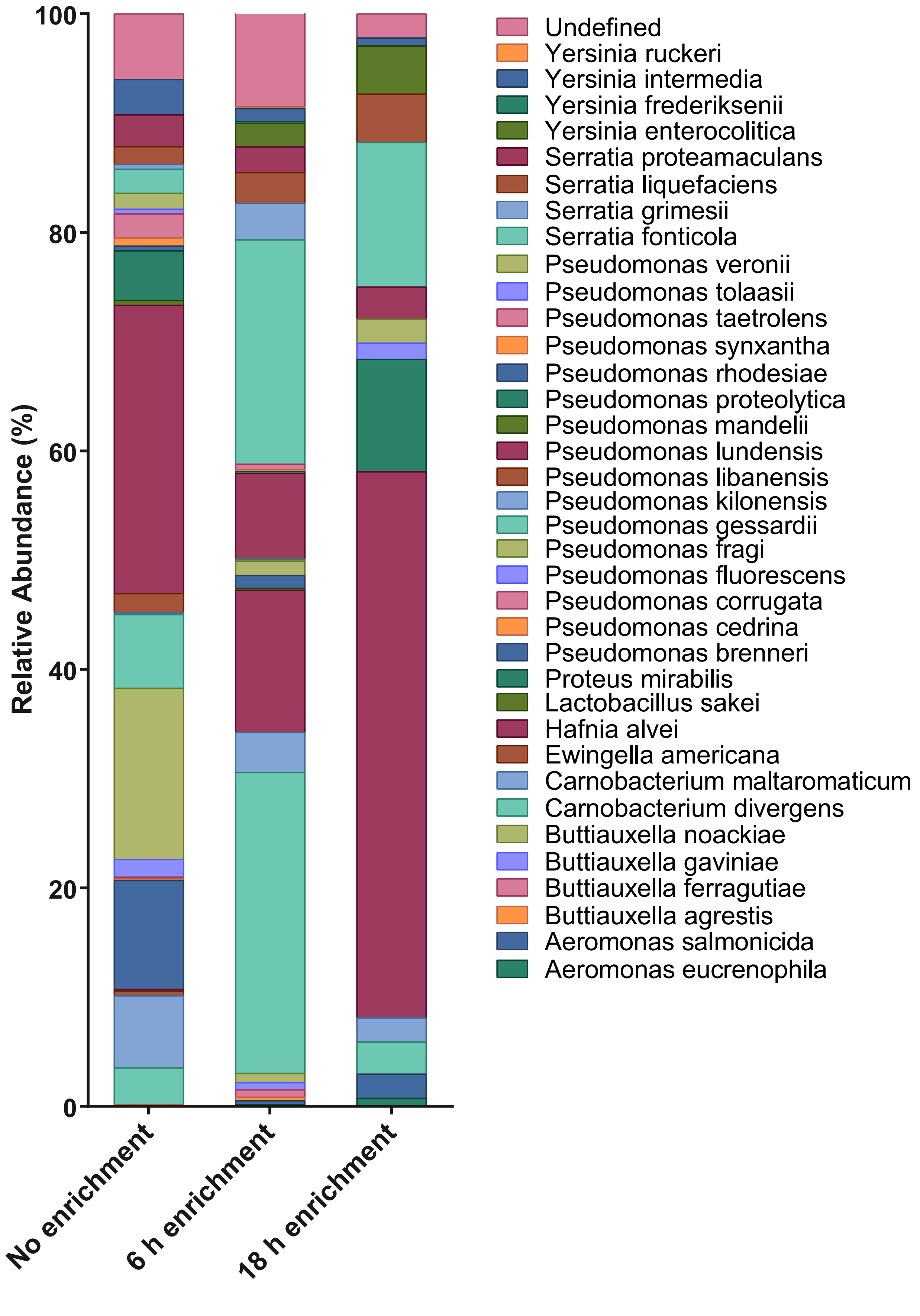

Supplementary Figure 1. **Relative abundances of species identified on retail chicken breast as determined by the Bruker Biotyper.** Taxa abundances of identified colonies at the species level are shown for each enrichment condition. Undefined: colonies with identification scores < 1.70 using the BDAL Bruker database (Version V9.0.0 8468).
