# Supplementary material for: Choice of DNA extraction method affects detection of bacterial taxa from retail chicken breast
Source: BMC Microbiol. 2022 Sep 30;22:230. doi: 10.1186/s12866-022-02650-7 (PMC9524001; doi:10.1186/s12866-022-02650-7)
Supplement: Supplementary file 2 — Additional file 2: Supplementary Figure 2. Microbial composition is altered by enrichment time. [file 12866_2022_2650_MOESM2_ESM.pdf]

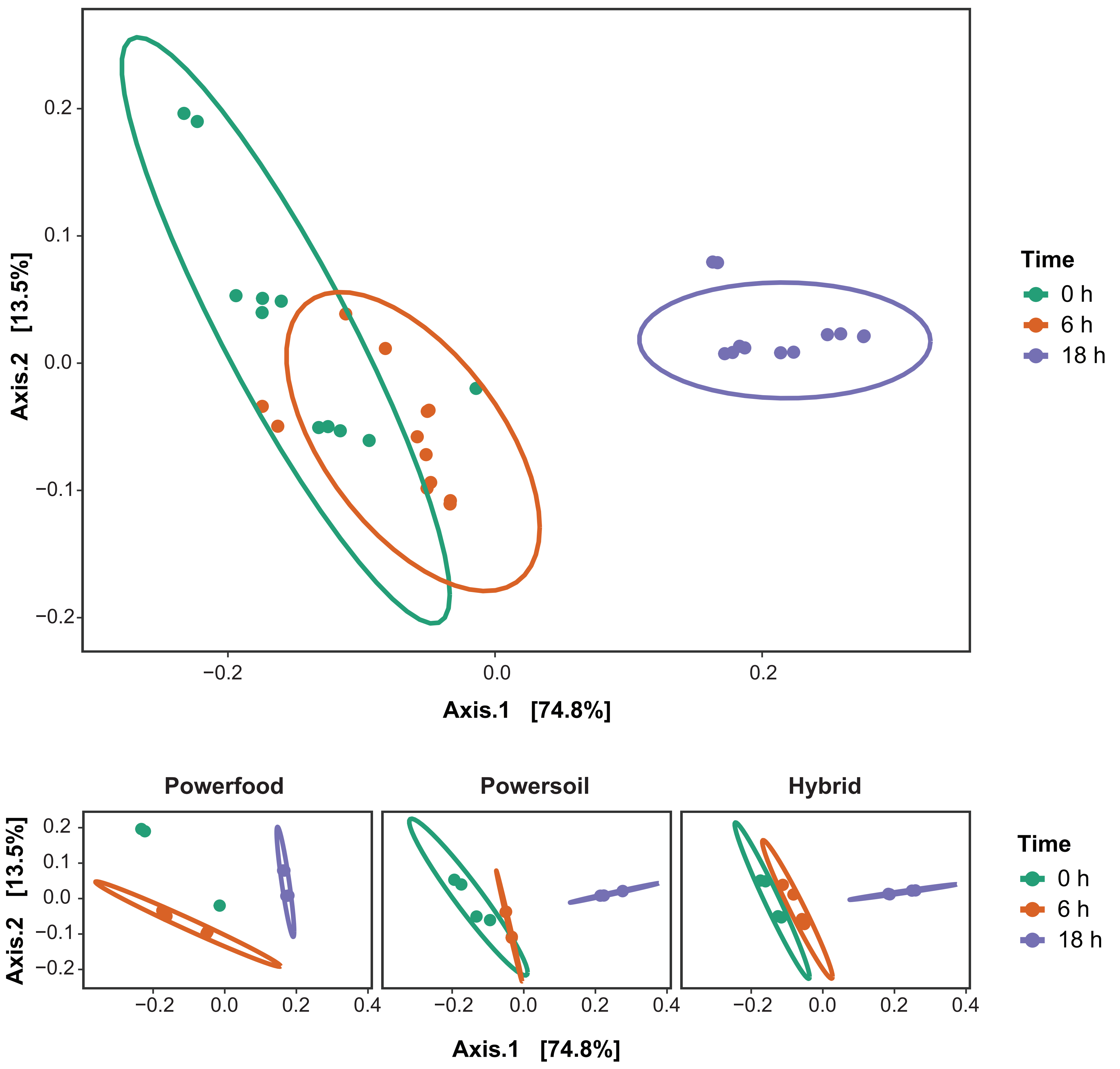

Supplementary Figure 2. **Microbial composition is altered by enrichment time.** Principal co-ordinate analysis of weighted Unifrac distances for all 16S samples (upper panel) and 16S samples by DNA extraction kit (lower panel). Ellipses represent 95% confidence regions.
